# Penile Dorsal Vein Rupture Identified by Emergency Department Ultrasound

**DOI:** 10.5811/cpcem.2020.10.49631

**Published:** 2020-12-07

**Authors:** Sean E. Scott, Robert Langenohl, Theodore Crisostomo-Wynne, Christopher Kang

**Affiliations:** Madigan Army Medical Center, Department of Emergency Medicine, Tacoma, Washington

**Keywords:** Penile fracture, emergency ultrasound

## Abstract

**Case Presentation:**

We present the case of a young male with high clinical suspicion of a penile fracture found to have dorsal vein rupture by emergency department point-of-care ultrasound. This false form of penile fracture was subsequently confirmed intraoperatively.

**Discussion:**

Penile fracture is a rare clinical entity that may be separated into true vs false penile fracture, with only true fracture requiring surgery. The images submitted here add to the sparse literature evidence that point-of-care ultrasound can be used to differentiate between these two clinical entities. Additionally, this case report highlights an opportunity for further research into and application of point-of-care ultrasound to the evaluation of suspected penile fractures.

## CASE PRESENTATION

**Image 1:**
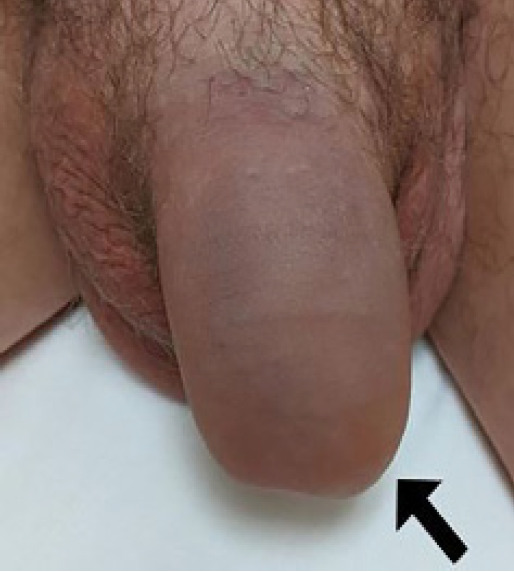
Patient’s penis with eggplant deformity (arrow) at presentation to the emergency department.

A 26-year-old man presented to the emergency department with penile pain and bruising after accidental trauma during intercourse one hour prior to arrival. He reported immediate pain and bruising with detumescence within minutes. Exam was revealing for eggplant deformity of the penis with soft dorsal ecchymosis and mass felt extending into the scrotum (Image 1).

## DISCUSSION

### Dorsal Vein Rupture

**Image 2:**
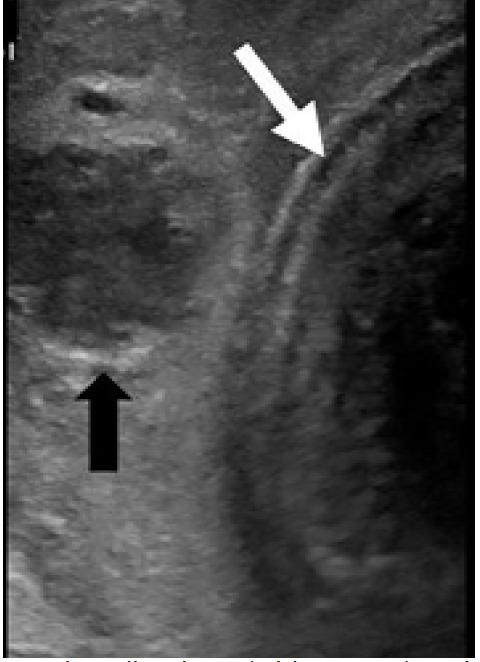
Intact tunica albuginea (white arrow) and likely ruptured vein with surrounding hematoma (black arrow).

**Image 3:**
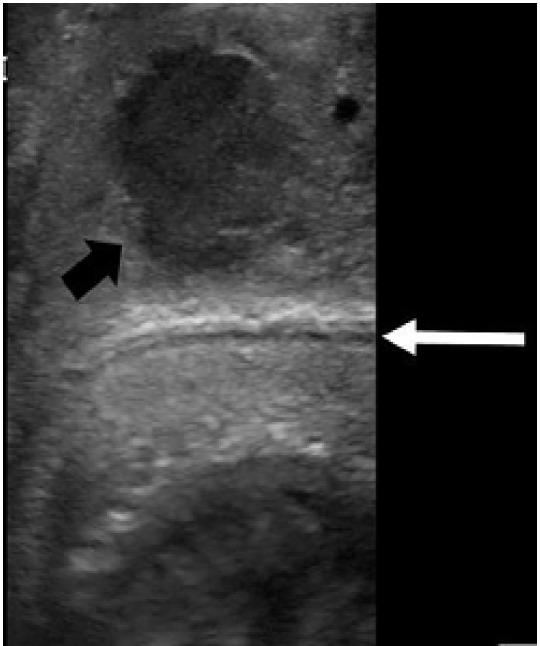
Intact tunica albuginea (white arrow) and likely ruptured dorsal vein with surrounding hematoma (black arrow).

This patient’s ultrasound images showed hematoma external to Buck’s fascia and intact tunica albuginea, suggestive of dorsal penile vessel injury (Images 2 and 3). This is a type of false penile fracture and requires only conservative treatment.^1^ True penile fracture, with disruption of the tunica albuginea, can be exceptionally difficult to distinguish from false penile fracture, has a worse prognosis, and requires emergency surgery.^1^ Along with history and physical exam, point-of-care ultrasound can help differentiate these entities.^2^ Delay of presentation greater than 24 hours of penile fracture has been linked to an increased rate of postoperative complications; so expeditious diagnosis is important to help reduce the time to intervention.^3^ Ultrasound can be an inexpensive, non-invasive, and often readily available means of investigation that may be performed at initial presentation without delay to surgery.^4^ False penile fracture is exceptionally rare, and is considered exceptionally difficult to distinguish from true penile fracture, often leading to surgery.^5^ Further research is necessary to help delineate the role of ultrasound in separating false from true penile fracture.

Surgical exploration confirmed a dorsal vein rupture with subsequent drainage of a large hematoma involving the dorsal vein and dartos layer dorsally. There were no acute complications. On follow-up the patient reported recovery of sexual function without curvature, but with mild residual pain to the incision site at six-week postoperative follow-up.

**CPC-EM Capsule**

What do we already know about this clinical entity?

Penile fracture is a rare clinical entity encountered following trauma to the erect penis and is typically managed surgically.

What is the major impact of the image(s)?

This image increases awareness of false penile fracture and the utility of ultrasound in evaluation for possible penile fracture.

How might this improve emergency medicine practice?

Increased use of point-of-care ultrasound may allow for improved accuracy in differentiating between true and false penile fracture and reduce the need for surgery.
